# The Expression of TMPRSS4 and Erk1 Correlates with Metastasis and Poor Prognosis in Chinese Patients with Gastric Cancer

**DOI:** 10.1371/journal.pone.0070311

**Published:** 2013-07-29

**Authors:** Zu-Yan Luo, Yuan-Yu Wang, Zhong-Sheng Zhao, Bo Li, Jun-Fa Chen

**Affiliations:** 1 Department of Radiology, Zhejiang Provincial People’s Hospital, Hangzhou, Zhejiang, People’s Republic of China; 2 Department of Gastrointestinal Surgery, Zhejiang Provincial People’s Hospital, Hangzhou, Zhejiang, People’s Republic of China; 3 Department of Pathology, Zhejiang Provincial People’s Hospital, Hangzhou, Zhejiang, People’s Republic of China; University of Alabama at Birmingham, United States of America

## Abstract

**Purpose:**

The present study investigated the clinical significance of transmembrane protease, serine 4(TMPRSS4) and extracellular signal-regulated kinases 1 (Erk1) in the development, progression and metastasis of gastric cancer.

**Methods:**

Immunohistochemistry was employed to analyze TMPRSS4 and Erk1 expression in 436 gastric cancer cases and 92 non-cancerous human gastric tissues.

**Results:**

Protein levels of TMPRSS4 and Erk1 were up-regulated in gastric cancer lesions compared with adjacent noncancerous tissues. High expression of TMPRSS4 correlated with age, size, Lauren’s classification, depth of invasion, lymph node and distant metastases, regional lymph node stage and TNM stage, and also with expression of Erk1. In stages I, II and III, the 5-year survival rate of patients with high TMPRSS4 expression was significantly lower than in patients with low expression. Further multivariate analysis suggests that up-regulation of TMPRSS4 and Erk1 were independent prognostic indicators for the disease, along with depth of invasion, lymph node and distant metastasis and TNM stage.

**Conclusions:**

Expression of TMPRSS4 in gastric cancer is significantly associated with lymph node and distant metastasis, high Erk1 expression, and poor prognosis. TMPRSS4 and Erk1 proteins could be useful markers to predict tumor progression and prognosis of gastric cancer.

## Introduction

Type II transmembrane serine proteases (TTSPs) have been recognized as a new subfamily of serine proteases with a proteolytic domain, a transmembrane domain, a short cytoplasmic domain and a stem region of variable length that contains modular structural domains[1]–[2]. Most TTSPs have been implicated in tumor development and progression, mainly based on their dysregulated expression. Transmembrane protease, serine 4(TMPRSS4) is a TTSP that is highly expressed in pancreatic, thyroid, lung and colorectal cancers[3]–[4]. TMPRSS4 induced invasiveness and epithelial-mesenchymal transition (EMT) by activating FAK signaling and Erk [5]. Extracellular signal-regulated kinase 1 (Erk1) is a member of the MAP kinase family and acts in a signaling cascade that regulates various cellular processes such as proliferation, differentiation, and cell cycle progression in response to a variety of extracellular signals [6]. The biological functions and clinical significance of TMPRSS4 and Erk1 in gastric cancer are not understood. The current study examined the expression of TMPRSS4 and Erk1 in 436 surgical specimens of gastric cancer by immunohistochemistry, to explore the possible correlation of TMPRSS4 and Erk1 expression with clinicopathological variables, and to determine the prognostic value of TMPRSS4 and Erk1 expression.

## Materials and Methods

### Archived Gastric Tissue Samples and Non-tumor Mucosa

Gastric cancer tissues were collected from gastrectomy specimens from 436 patients (311 male, 125 female; median age 60.0 years, range 30–91) from the Department of Surgery, Zhejiang Provincial People’s Hospital, from January 1998 to January 2004. Tissues had been formalin-fixed, paraffin-embedded, and diagnosed clinically and histopathologically at the Departments of Gastrointestinal Surgery and Pathology. All patients had follow-up records for >5 years. The follow-up deadline was December 2008. The survival time was calculated from the date of surgery to the follow-up deadline or date of death, which was caused mainly by carcinoma recurrence or metastasis. Ninety-two non-cancerous human gastric tissues were obtained from gastrectomy of adjacent gastric cancer margins >5 cm. Routine chemotherapy was given to the patients with advanced-stage disease after operation, but no radiation treatment was administered to any of the patients included in our study.

### Tissue Microarray Analysis

Blocks that contained a total of 436 tumour tissue samples and 92 samples of normal gastric mucosa were prepared as described previously [7]–[8]. Core tissue biopsies (2 mm in diameter) were taken from individual paraffin-embedded gastric tumors (donor blocks) and arranged in recipient paraffin blocks (tissue array blocks) using a trephine. Since it has been proven that staining results obtained from different intratumoral areas in various tumors correlate well [9], a core was sampled in each case. An adequate case was defined as a tumor occupying >10% of the core area [10]. Each block contained more than three internal controls that consisted of non-neoplastic gastric mucosa. Four-micrometer-thick sections were cut from each tissue array block, deparaffinized and dehydrated.

The project was approved by the ethics committee of Zhejiang Provincial People’s Hospital and written consent were obtained from all the participants.

### Immunohistochemistry

Immunohistochemical analysis [11]–[12] was performed to study altered protein expression in 92 non-cancerous human gastric tissue samples and 436 human gastric cancer tissues. In brief, slides were baked at 60°C for 2 h followed by deparaffinization with xylene, and rehydrated. The sections were submerged in EDTA antigenic retrieval buffer and microwaved for antigen retrieval, after which they were treated with 3% hydrogen peroxide in methanol to quench endogenous peroxidase activity, followed by incubation with 1% bovine serum albumin to block non-specific binding. Sections were incubated with rabbit anti-TMPRSS4 (ProteinTech Group, Inc) and rabbit anti-Erk1(Cell Signaling Technology), overnight at 4°C. Normal goat serum was used as a negative control. After washing, tissue sections were treated with secondary antibody. Tissue sections were then counterstained with hematoxylin, dehydrated, and mounted.

### Evaluation of Results

TMPRSS4 and Erk1 were stained as buffy colored in the cytoplasm and nucleus. The degree of immunostaining was reviewed and scored independently by two observers based on the intensity of staining [11], [13], [14]. Staining intensity was graded according to the following criteria: 0 (no staining), 1 (weak staining = light yellow), 2 (moderate staining = yellow brown), and 3 (strong staining = brown). Moderate and strong staining were used to define tumors with high TMPRSS4 or Erk1 expression, and no and weak staining were used to indicate low TMPRSS4 or Erk1 expression.

### Statistical Analysis

All statistical analyses were performed using SPSS 12.0 software. Measurement data were analyzed using Student’s *t* test, while categorical data were studied using the *χ* ^2^ or Fisher exact test. Survival curves were estimated using the Kaplan–Meier method, and the log-rank test was used to calculate differences between the curves. Multivariate analysis using the Cox proportional hazards regression model was performed to assess the prognostic values of protein expression. Correlation coefficients between protein expression and clinicopathological findings were estimated using the Pearson correlation method. Statistical significance was set at *P*<0.05.

## Resuts

### Expression of TMPRSS4 in Archived Gastric Tissue Samples and Non-tumor Mucosa

**Figure 1:**
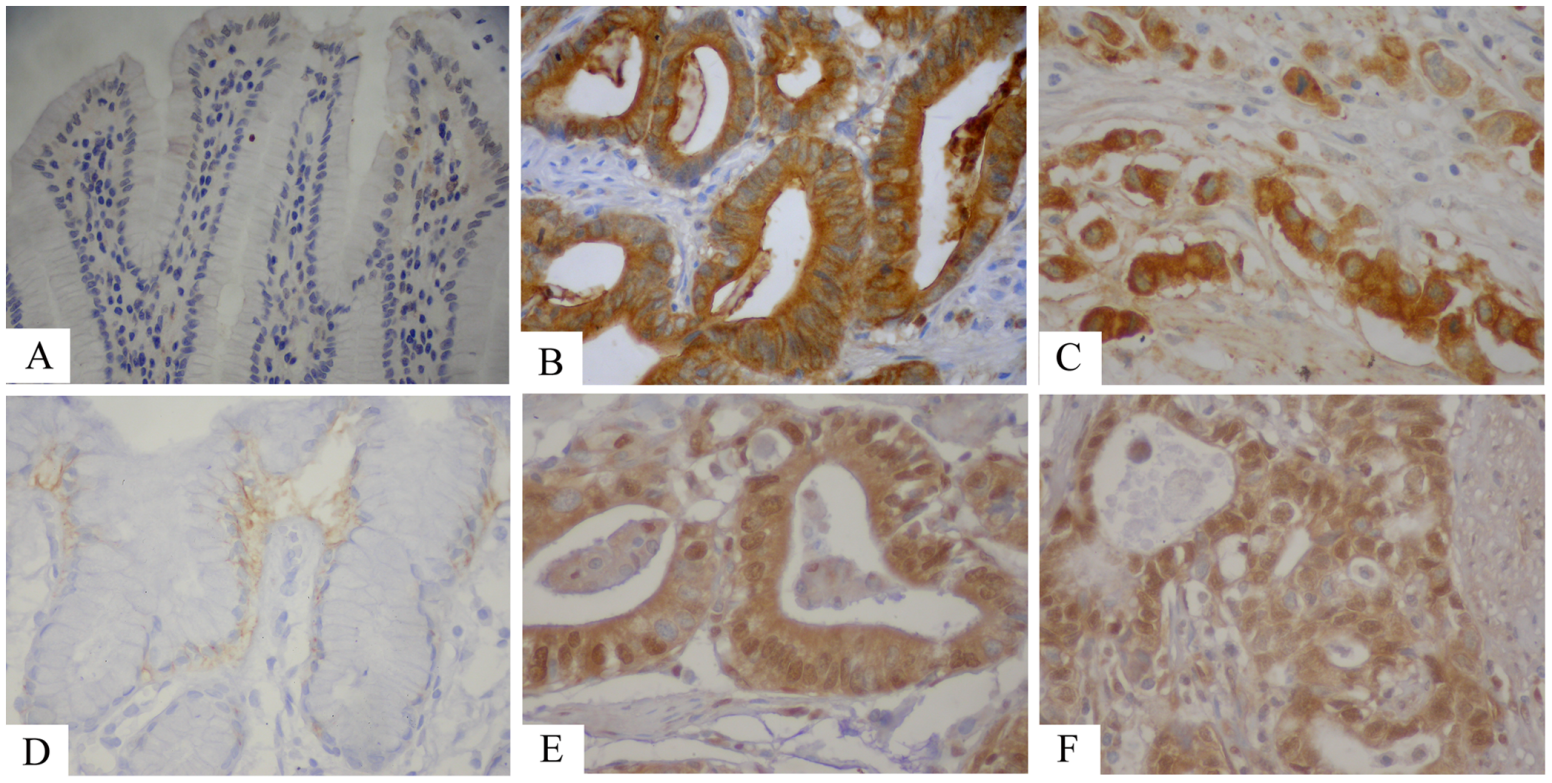
Immunohistochemical staining for TMPRSS4 and Erk1 in gastric cancer lesions and noncancerous tissues. **A** TMPRSS4 negative in noncancerous tissues, magnification×400. **B** TMPRSS4 was highly expressed in moderately differentiated adenocarcinoma, magnification×400. **C** TMPRSS4 was highly expressed in poorly differentiated adenocarcinoma, magnification×400, respectively. **D** Erk1 negative in noncancerous tissues, magnification×400. **E** Erk1 was highly expressed in moderately differentiated adenocarcinoma, magnification×400. **F** Erk1 was highly expressed in poorly differentiated adenocarcinoma, magnification×400.

TMPRSS4 protein was detected in 14 of 92 (15.22%) human non-tumor mucosa samples, and all samples expressed the protein at a low level. TMPRSS4 protein was detected in 245 of 436 (56.19%) cases of human gastric cancer, high expression of TMPRSS4 protein was detected in 203 (46.56%) tumors. TMPRSS4 was localized mainly in the cytoplasm or nucleus of primary cancer (Figure 1A, B, and C).

### Expression of Erk1 in Archived Gastric Tissue Samples and Non-tumor Mucosa

Erk1 protein was detected in 16 of 92 (17.39%) human non-tumor mucosa samples, and all samples expressed the protein at a low level. Erk1 protein was detected in 231 of 436 (52.98%) cases of human gastric cancer, high expression of Erk1 protein was detected in 173(39.68%) tumors. Erk1 was localized mainly in the cytoplasm or nucleus of primary cancer (Figure 1D, E and F).

### Correlation between TMPRSS4 Up-regulation and Clinical Features of Gastric Cancer

High expression of TMPRSS4 correlated with age, size, Lauren’s classification, depth of invasion, lymph node and distant metastases, regional lymph node stage and TNM stage (*P*<0.05) (Table 1). TMPRSS4 expression did not correlate with sex, tumor location, differentiation, or histological classification (*P*>0.05) (Table 1).

**Table 1:** Correlation between TMPRSS4 expression and clinicopathological features of gastric cancer.

Clinical parameters	TMPRSS4 expression			
	low	high	t/χ^2^/r	*P*
Age(yrs)	56.58±11.14	61.89±12.60	4.667	0.01
Gender			0.354	0.552
Male	169(54.3%)	142(45.7%)		
Female	64(51.2%)	61(48.8%)		
Location			4.205	0.122
Proximal	24(43.6%)	31(56.4%)		
Middle	83(50.9%)	80(49.1%)		
Distal	126(57.8%)	92(42.2%)		
Size			37.00	0.00
<5 cm	168(65.6%)	88(34.4%)		
≥5 cm	65(36.1%)	115(63.9%)		
Lauren classification			141.0	0.001
Intestinal	181(81.2%)	42(18.8%)		
Diffuse	52(24.4%)	161(75.6%)		
Histology			1.956	0.582
Papillary adenocarcinoma	9(56.2%)	7(43.8%)		
Tubular adenocarcinoma	178(54.6%)	148(45.4%)		
Mucinous adenocarcinoma	12(41.4%)	17(58.6%)		
Signet-ring cell carcinoma	34(52.3%)	31(47.7%)		
Histologic differentiation			7.33	0.062
Well	11(84.6%)	2(15.4%)		
Moderately	74(57.8%)	54(42.2%)		
Poorly	147(50.2%)	146(49.8%)		
Others	1(50.0%)	1(50.0%)		
Invasion depth			81.99	0.001
T1	54(94.7%)	3(5.3%)		
T2	75(68.8%)	34(31.2%)		
T3	101(41.4%)	143(58.6%)		
T4	3(11.5%)	23(88.5%)		
TNM Stages			180.6	0.00
I	86(95.6%)	4(4.4%)		
II	83(79.8%)	21(20.2%)		
III	60(34.7%)	113(65.3%)		
IV	4(5.8%)	65(94.2%)		
Vessel invasion			95.39	0.001
No	148(80.9%)	35(19.1%)		
Yes	85(33.6%)	168(66.4%)		
Lymphatic metastasis			102.8	0.001
No	140(84.3%)	26(15.7%)		
Yes	93(34.4%)	177(65.6%)		
Regional lymph nodes			136.4	0.001
PN0	140(84.3%)	26(15.7%)		
PN1	69(50.7%)	67(49.3%)		
PN2	23(23.2%)	76(76.8%)		
PN3	1(2.9%)	34(97.1%)		
Distant metastasis			62.65	0.001
No	229(61.1%)	146(38.9%)		
Yes	4(6.6%)	57(93.4%)		

### Correlation between Erk1 Up-regulation and Clinical Features of Gastric Cancer

High expression of Erk1 correlated with age, tumor location, size, depth of invasion, differentiation, Lauren’s classification, lymph node and distant metastases, regional lymph node stage and TNM stage (*P*<0.05) (Table 2). Erk1 expression did not correlate with sex, or histological classification (*P*>0.05) (Table 2).

**Table 2:** Correlation between Erk1 expression and clinicopathological features of gastric cancer.

Clinical parameters	Erk1 expression			
	low	high	t/χ^2^/r	*P*
Age(yrs)	57.68±11.46	61.13±12.83	2.93	0.004
Gender			0.27	0.603
Male	190(61.1%)	121(38.9%)		
Female	73(58.4%)	52(41.6%)		
Location			8.129	0.017
Proximal	24(43.6%)	31(56.4%)		
Middle	98(60.1%)	65(39.9%)		
Dist1al	141(64.7%)	77(35.3%)		
Size			32.29	0.001
<5 cm	183(71.5%)	73(28.5%)		
≥5 cm	80(44.4%)	100(55.6%)		
Lauren classification			169.5	0.001
Intestinal	201(90.1%)	22(9.9%)		
Diffuse	62(29.1%)	151(70.9%)		
Histology			2.876	0.411
Papillary adenocarcinoma	11(68.8%)	5(31.2%)		
Tubular adenocarcinoma	202(62.0%)	124(38.0%)		
Mucinous adenocarcinoma	15(51.7%)	14(48.3%)		
Signet-ring cell carcinoma	35(53.8%)	30(46.2%)		
Histologic differentiation			8.093	0.044
Well	12(92.3%)	1(7.7%)		
Moderately	83(64.8%)	45(35.2%)		
Poorly	167(57.0%)	126(43.0%)		
Others	1(50.0%)	1(50.0%)		
Invasion depth			69.27	0.001
T1	55(96.5%)	2(3.5%)		
T2	81(74.3%)	28(25.7%)		
T3	122(50.0%)	122(50.0%)		
T4	5(19.2%)	21(80.8%)		
TNM Stages			153.0	0.001
I	84(93.3%)	6(6.7%)		
II	91(87.5%)	13(12.5%)		
III	79(45.7%)	94(54.3%)		
IV	9(13.0%)	60(87.0%)		
Vessel invasion			74.84	0.001
No	154(84.2%)	29(15.8%)		
Yes	109(43.1%)	144(56.9%)		
Lymphatic metastasis			97.06	0.001
No	149(89.8%)	17(10.2%)		
Yes	114(42.2%)	156(57.8%)		
Regional lymph nodes			136.6	0.001
PN0	149(89.8%)	17(10.2%)		
PN1	81(59.6%)	55(40.4%)		
PN2	30(30.3%)	69(69.7%)		
PN3	3(8.6%)	32(91.4%)		
Distant metastasis			57.18	0.001
No	253(67.5%)	122(32.5%)		
Yes	10(16.4%)	51(83.6%)		

### Correlation between TMPRSS4 Expression and Prognosis

**Figure 2:**
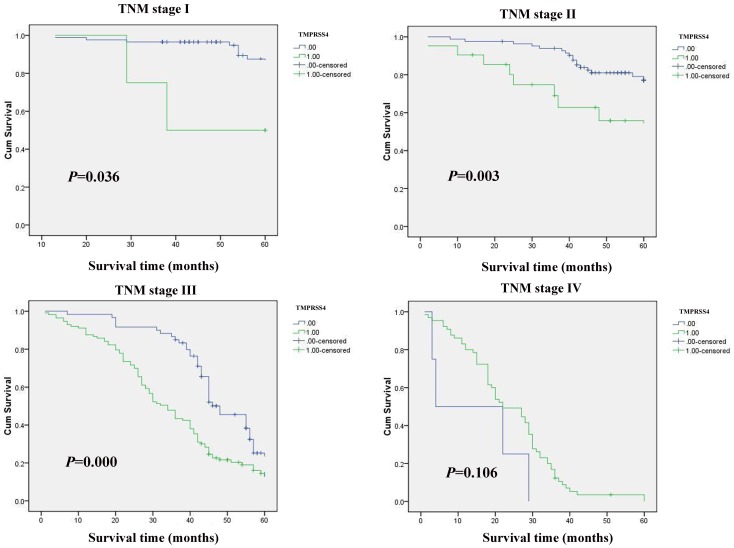
Kaplan-Meier curves with univariate analyses (log-rank) for patients with low TMPRSS4 expression versus high TMPRSS4 expression tumors. 0: negative, 1: positive.

In stage I, II and III tumors, the 5-year survival rate in patients with high expression of TMPRSS4 was significantly lower than that in patients with low expression (*P*<0.05) (Figure 2). In stage IV tumors, the expression of TMPRSS4 did not correlate with the 5-year survival rate (*P*>0.05) (Figure 2).

### Correlation between Erk1 Expression and Prognosis

**Figure 3:**
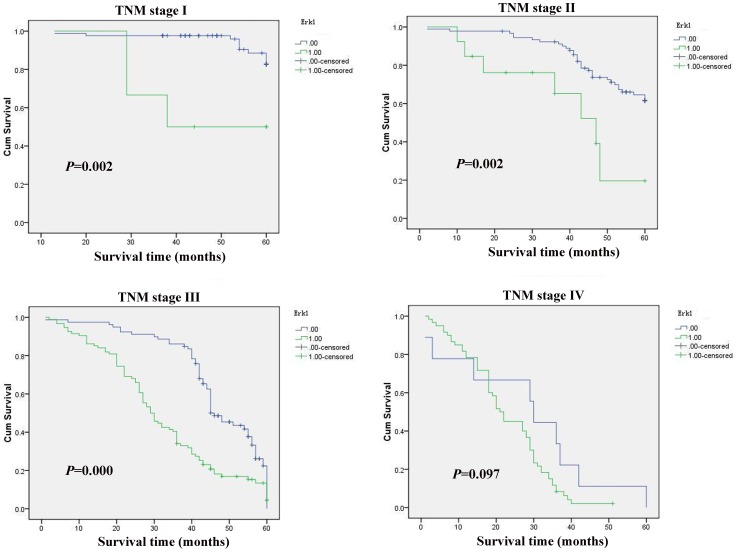
Kaplan-Meier curves with univariate analyses (log-rank) for patients with low Erk1 expression versus high Erk1 expression tumors in different TNM stage. 0: negative, 1: positive.

In stage I, II and III tumors, the 5-year survival rate in patients with high expression of Erk1 was significantly lower than that in patients with low expression (*P*<0.05) (Figure 3). In stage IV tumors, the expression of Erk1 did not correlate with the 5-year survival rate (*P*>0.05) (Figure 3).

### Multivariate Analysis of Clinicopathological Parameters and Prognosis

The factors with possible prognostic effects in gastric carcinoma were analyzed by Cox regression analysis. The study revealed that lymph node and distant metastases (*P* = 0.005), TNM stage (*P* = 0.005), expression of TMPRSS4 (*P* = 0.000) and Erk1 (*P* = 0.000) were independent prognostic factors of patients with gastric carcinoma. However, age, sex, tumor location and size, histological classification, tumor differentiation, invasion depth, Lauren’s classification, and regional lymph node stage had no prognostic value.

### Association among Expression of TMPRSS4 and Erk1

**Figure 4:**
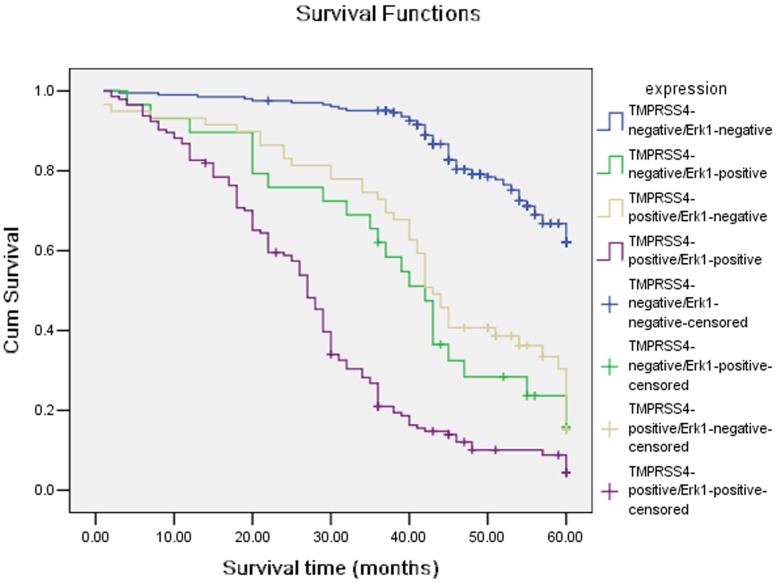
Kaplan-Meier curves with univariate analyses (log-rank) for patients withTMPRSS4-positive/Erk1-negative, TMPRSS4-positive/Erk1-positive, TMPRSS4-negative/Erk1-positive and TMPRSS4-negative/Erk1-negative gastric patients.

Two hundred and four gastric cancer cases had low expression of both TMPRSS4 and Erk1, and one hundred and forty-four gastric cancer cases had high expression of both TMPRSS4 and Erk1. There was significant correlation between TMPRSS4 and Erk1 (χ^2^ = 155.1; P = 0.0001) (Table 3). We also detected the outcomes of TMPRSS4-positive/Erk1-negative, TMPRSS4-positive/Erk1-positive, TMPRSS4-negative/Erk1-positive and TMPRSS4-negative/Erk1-negative gastric patients, and we found that there was a significant difference in overall comparisons (χ^2^ = 256.9; P = 0.000, Figure 4). The mean survival time were 42.8±2.23, months for TMPRSS4(+)/Erk1(−) group, 27.9±1.27 months for TMPRSS4(+)/Erk1(+), 54.7±0.72 months for TMPRSS4(−)/Erk1(−), 39.2±3.19 months for TMPRSS4(−)/Erk1(+), respectively.

**Table 3:** Correlation between TMPRSS4 and Erk1 in gastric cancer.

	Erk1		χ^2^	*P* value
	Positive (%)	Negative (%)		
**TMPRSS4**				
Positive (%)	144 (70.9%)	59 (29.1%)		
Negative (%)	29 (12.4%)	204 (87.6%)	155.1	0.0001

## Discussion

Of the many molecules involved in the carcinogenesis and development of human tumors, proteases have been extensively studied as important participants in the carcinogenesis of many tumors. Recently, much attention has been focused on the role of type II transmembrane serine proteases (TTSPs) during tumor development [15]–[16]. TTSPs are members of the family of cell surface-associated proteases that mediate a variety of normal cellular functions as well as tumor invasion and metastasis.

TMPRSS4 is a TTSP that has been known to be upregulated in several cancers, particularly in pancreatic and thyroid cancers [4], [17], and the expression of TMPRSS4 has been correlated with the metastatic potential of pancreatic cancer [3]. TMPRSS4 is necessary and sufficient for the human lung cancer cell line NCI-H322, the colon adenocarcinoma cell line Colo205, and the colorectal cancer cell line HCT15 to accomplish migration and invasion [18]. The study revealed that the expression of TMPRSS4 in gastric cancer lesions were closely associated with the age, size of tumor, location of tumor, depth of invasion, vessel invasion, lymph node and distant metastasis and TNM stage. The expression of TMPRSS4 was found to significantly correlate with the prognosis of gastric cancer. In stages I, II and III, the 5-year survival rates of patients with high expression of TMPRSS4 was significantly lower than those in patients with low expression. In stage IV, TMPRSS4 expression did not correlate with the 5-year survival rate. Further multivariate analysis suggested that the depth of invasion, lymph node and distant metastasis, TNM stage, and up-regulation of TMPRSS4 were independent prognostic indicators for gastric cancer. Overexpression of TMPRSS4 has a critical role in radiation-induced long-term dissemination and metastasis of residual hepatocellular carcinoma by facilitating epithelial-mesenchymal transition (EMT) [19]. Expression of TMPRSS4 in tumours with squamous cell carcinoma (SCC) histology was found to be significantly higher than those with adenocarcinoma (AC) histology. Kaplan-Meier curves demonstrated that high levels of TMPRSS4 were significantly associated with reduced overall survival in the patients with SCC histology, whereas no correlation was found for the AC histology [20].

Erk1 involved in the carcinogenesis through regulating various cellular processes such as proliferation, differentiation, and cell cycle progression in response to a variety of extracellular signals. Hepatoma-derived growth factor is involved in the gastric carcinogenesis process and promotes proliferation and metastasis via Erk1/2 activation [21]. ERK1 as an important mediator of lysophosphatidic acid signaling leading to upregulation of sphingosine kinase 1 (SphK1) and point to SphK1 and sphingosine-1-phosphate production as potential therapeutic targets in gastric cancer [22]. The interaction between FVIIa and TF induces protease-activated receptor 2 activation, thereby triggers the ERK1/2 and IκB-α/NF-κB signal transduction pathway to regulate the gene expression of IL-8, TF, and caspase-7, and ultimately promotes SW620 cell proliferation and migration [23]. The expression of Erk1 in gastric cancer and its clinical significance are not understood. The current study was to examine the expression of Erk1 in 436 surgical specimens of gastric cancer by immunohistochemistry. The study showed that high expression of Erk1 protein was detected in 173(39.68%) tumors. High expression of Erk1 correlated with age, tumor location, size, depth of invasion, differentiation, Lauren’s classification, lymph node and distant metastases, regional lymph node stage, TNM stage and poor prognosis.

We further examined TMPRSS4 expression in gastric cancer specimens and its correlation with Erk1 expression. We found a positive correlation between TMPRSS4 and Erk1 expression, suggesting that TMPRSS4 may be involved in the carcinogenesis, progression and metastasis of gastric cancer through promotion of cell proliferation by Erk1 activation.
